# Short-term vs. long-term heart rate variability in ischemic cardiomyopathy risk stratification

**DOI:** 10.3389/fphys.2013.00364

**Published:** 2013-12-13

**Authors:** Andreas Voss, Rico Schroeder, Montserrat Vallverdú, Steffen Schulz, Iwona Cygankiewicz, Rafael Vázquez, Antoni Bayés de Luna, Pere Caminal

**Affiliations:** ^1^Department of Medical Engineering and Biotechnology, University of Applied SciencesJena, Germany; ^2^Biomedical Engineering Research Centre, Universitat Politècnica de CatalunyaBarcelona, Spain; ^3^Department of Electrocardiology, Sterling Memorial University HospitalLodz, Poland; ^4^Servicio de Cardiología, Puerta del Mar University HospitalCádiz, Spain; ^5^Institut Català Ciències Cardiovasculars, Hospital Sant PauBarcelona, Spain

**Keywords:** risk stratification, heart rate variability, short-term, long-term, daytime, nighttime, nonlinear dynamics, ischemic cardiomyopathy

## Abstract

In industrialized countries with aging populations, heart failure affects 0.3–2% of the general population. The investigation of 24 h-ECG recordings revealed the potential of nonlinear indices of heart rate variability (HRV) for enhanced risk stratification in patients with ischemic heart failure (IHF). However, long-term analyses are time-consuming, expensive, and delay the initial diagnosis. The objective of this study was to investigate whether 30 min short-term HRV analysis is sufficient for comparable risk stratification in IHF in comparison to 24 h-HRV analysis. From 256 IHF patients [221 at low risk (IHF_LR_) and 35 at high risk (IHF_HR_)] (a) 24 h beat-to-beat time series (b) the first 30 min segment (c) the 30 min most stationary day segment and (d) the 30 min most stationary night segment were investigated. We calculated linear (time and frequency domain) and nonlinear HRV analysis indices. Optimal parameter sets for risk stratification in IHF were determined for 24 h and for each 30 min segment by applying discriminant analysis on significant clinical and non-clinical indices. Long- and short-term HRV indices from frequency domain and particularly from nonlinear dynamics revealed high univariate significances (*p* < 0.01) discriminating between IHF_LR_ and IHF_HR_. For multivariate risk stratification, optimal mixed parameter sets consisting of 5 indices (clinical and nonlinear) achieved 80.4% AUC (area under the curve of receiver operating characteristics) from 24 h HRV analysis, 84.3% AUC from first 30 min, 82.2 % AUC from daytime 30 min and 81.7% AUC from nighttime 30 min. The optimal parameter set obtained from the first 30 min showed nearly the same classification power when compared to the optimal 24 h-parameter set. As results from stationary daytime and nighttime, 30 min segments indicate that short-term analyses of 30 min may provide at least a comparable risk stratification power in IHF in comparison to a 24 h analysis period.

## Introduction

Heart failure (HF) is a major escalating public health problem worldwide, particularly in industrialized countries with aging populations, being associated with both high morbidity and mortality (McMurray and Stewart, 2000; Felker, 2012). Generally, HF is a final manifestation of most cardiac diseases and is clinically recognized by a multitude/complexity of signs and symptoms caused by complex circulatory and neurohormonal responses to structural and/or functional cardiac dysfunction (Guindo et al., 1997; Rohini et al., 2012). Typically, HF is characterized by impaired ventricular filling and/or ventricular emptying into the aorta and pulmonary artery. Considering the aetiology of the left ventricular dysfunction, patients with HF may be divided into patients with ischemic HF and patients with nonischemic HF (Kitsios and Zintzaras, 2007). As one of the most common cardiovascular diseases, clinically identified HF affects 0.3–2% of the general population. In the population older than 65 years, the prevalence of HF is larger than 100 cases/1000 individuals. The five-year mortality after initial diagnosis of HF is approximately 60–70% (McMurray and Stewart, 2000; Bleumink et al., 2004; Strömberg and Jaarsma, 2008). An early risk prediction of HF may contribute in assessing the prognosis of HF and in finding an adequate medical treatment or finding the optimal timing for either prophylactic defibrillator implantation or, at worst, a cardiac transplantation. However, even today there are still no generally accepted indications identifying HF patients with an increased risk of sudden cardiac death (SCD). Thus, the identification of HF patients at risk still remains an important key issue in clinical decision making (Goldberger et al., 2008; Cygankiewicz et al., 2009; Saha and Goldberger, 2012). In recent decades, the need to risk-stratify patients for targeted therapy has encouraged the search for Holter-based risk predictors of increased risk (Stein and Reddy, 2005). Although several risk stratification studies investigated the usefulness of primarily univariate analysis of heart rate variability (HRV) using linear and non-linear methods, no satisfactory contribution to risk prediction in HF patients has been achieved. HRV analysis based on beat-to-beat dynamics may reveal specific changes of heart rate time series, allowing for the assessment of the role of autonomic nervous system (ANS) fluctuations in both healthy subjects and those with cardiovascular disease (Task Force, 1996). Conventional HRV analysis with linear methods from time and frequency domain is most often used for assessing the phase and average magnitude of changes between successive heartbeats. Due to very complex behavior in generating the heart rate caused by different components of the intrinsic system's dynamics, and especially by the nonlinear interplay of different physiological control loops, linear methods are not adequate to fully describe such a complex system. Several nonlinear HRV methods such as different fractal scaling analysis, power law analysis, complexity analysis, symbolic dynamics methods and heart rate turbulence analysis have been studied for various diseases (Huikuri et al., 2009; Voss et al., 2009). In a previous study (Voss et al., 2010b), the potential of long-term (24-h) nonlinear indices of HRV for enhanced risk stratification in patients with ischemic heart failure (IHF) was demonstrated. However, long-term analyses are time-consuming and expensive, and they delay the initial diagnosis. To reduce this drawback, the objective of this study was to investigate whether a 30 min short-term HRV analysis (symbolic dynamics indices require at least 1500 samples—that means approximately 20–30 min) is sufficient for comparable risk stratification in IHF as compared to 24 h HRV analysis. To achieve this goal, multivariate optimal parameter sets consisting of clinical and/or linear and/or nonlinear HRV indices were calculated, analysing (a) 24 h ECGs and (b) the first 30 min ECG segments. Furthermore, due to the circadian chronobiological pattern of HRV (c) the 30 min most stationary day ECG segments and (d) the 30 min most stationary night ECG segments were also analyzed. Finally, optimal parameter sets determined for (a) through (d) were compared with each other.

## Materials and methods

### Data acquisition and pre-processing

During the Spanish prospective multicenter MUSIC2 study (Muerte Subita en Insuficiencia Cardiaca or Sudden Death in Heart Failure), 24 h Holter ECGs (three orthogonal leads, 200 Hz sampling frequency, sensitivity threshold of 10μV) were recorded (ELA Medical SyneflashTM MMC, Plymouth, Minnesota, USA) from patients suffering from IHF. All digitized ECGs and patients' clinical data were stored together in a database. From all raw data records, time series of heart rate (tachograms) consisting of successive beat-to-beat intervals (BBI) were extracted (ELA Medical Synetec™). Afterwards, all times series were filtered by an adaptive filter algorithm which replaces and interpolates ventricular premature beats and artefacts generating normal-to-normal (NN) interval time series (Wessel et al., 2000). For the performed HRV analysis, NN interval time series of 24 h and 30 min lengths were investigated:

– 24 h NN interval time series,
– The first 30 min NN interval time series segments,
– The 30 min most stationary day NN interval time series segments,
– The 30 min most stationary night NN interval time series segments.

Since ECG recordings were started at different times (latest start time: 15:28 h and earliest stop time: 04:56 h), the NN interval time series for day and night phases were aligned in terms of a common start and stop time. Daytime (4 h) was defined from 16:00 to 20:00 h and nighttime (4 h) from 24:00 to 04:00 h.

#### Stationarity

An additional criterion was stationarity of the 30 min day and night segments, since some of the applied methods (e.g., frequency domain analysis) require stationarity conditions. Due to the fact that variations in time series can be caused by environmental stimuli (various external factors) or essentially by the dynamics of the nonlinear system (Peng et al., 1995) turn into a problem of differentiating these variations. Stationarity requires that statistical properties such as mean and standard deviation of the investigated NN interval time series remain the same throughout the investigated time segment. If the stationarity requirements are not met, as is for most complex, not random physiological signals, then an impact of trends with change on the mean of the time series have to be considered in the interpretation of the results (Seely and Macklem, 2004; Voss et al., 2009). By means of stationary NN interval time segments, a possible bias on our final results will be excluded or at least minimized. In accordance with Schulz et al. (Schulz et al., 2011), we therefore extracted and used only 30 min NN time segments for daytime and nighttime hours, thereby fulfilling the pre-selection criterion for most stationary segments.

The most stationary segments were extracted as:

1. Linear interpolation of the 4 h NN interval time series (day or night extracted NN segments) with a sampling frequency of 4 Hz.
2. Segmentation of the interpolated 4 h NN interval time series in 29 min overlapping windows (shift = 1 min) of 30 min window length.
3. Calculation of following local indices for each window *w*:
  - Mean value (meanNN30 min_*w*_, [ms]),
  - Standard deviation (sdNN30 min_*w*_, [ms]),
  - Coefficient of variation (cvNN30 min_*w*_ = sdNN30 min_*w*_/(meanNN30 min_*w*_)^3^, [1/ms^2^]).
4. Extracting the most stationary NN interval segment with the smallest cvNN30 min.

### Methods of heart rate variability analysis

For HRV analysis, several linear and nonlinear approaches were proposed. Traditional linear time and frequency domain analysis methods assess the phase and the average magnitude of time series changes between consecutive beats, whereas nonlinear ones quantify the signal properties (dynamic and structural features) (Voss et al., 2009, 2011).

#### Time and frequency domain

HRV were quantified by calculating standard indices from the time domain (TD) and frequency domain (FD) of each tachogram according to the recommendations provided by the Task Force of the European Society of Cardiology (Task Force, 1996).

The following TD indices were estimated:

– meanNN = mean value of NN interval time series [ms],
– sdNN = standard deviation of NN interval time series [ms] and
– rmssd = square root of the mean squared differences of successive NN intervals [ms].

The fast Fourier transform with a Blackman Harris window function was applied to estimate the power spectra of the NN interval time series. For equidistant time series, all tachograms were linearly interpolated and subsequently resampled (2 Hz).

Subsequently, the following FD indices were determined using the power spectra:

– LF/HF = ratio between low-frequency (0.04–0.15 Hz) power and high-frequency (0.15–0.4 Hz) power,
– VLF/*P* = normalized very low-frequency power (≤0.04 Hz),
– *P* = total power of the spectra [ms^2^].

#### Classical symbolic dynamics

In 1993, Voss et al. (1993, 1996); Kurths et al. (1995) introduced the classical symbolic dynamics (*SD*) approach in HRV analysis. SD is based on coarse-graining the original time series by applying a defined number of symbols; it has been proven sufficient for the investigation of complex systems and in describing dynamic properties within time series. The first step of the classical SD algorithm involves the transformation of NN interval time series into strings of symbols using the alphabet *A* = {0, 1, 2, 3} according to transformation rules:

$$\begin{array}{l} \;0:\;\mu \;<{\text{ NN}}_{n}\;<=\;(1+\alpha )\times \mu \\ \;1:\;(1+\alpha )\times \mu \;<{\text{ NN}}_{n}\;<\;\infty \\ 2:\;(1-\alpha )\times \mu \;<{\text{ NN}}_{n}\;<=\;\mu \\ \;3:\;0\;<{\text{ NN}}_{n}\;<=\;(1-\alpha )\times \mu . \end{array}$$

Here μ is the mean of all NN intervals, α is a special scaling parameter set to 0.1 and NN*n* is the NN interval at the time point *n*. Afterwards, words consisting of three successive symbols were achieved from the symbol string resulting in 4^3^ = 64 different word types. A histogram containing the probability distribution of each possible word type (000, 001, …, 333) within the symbol sequence was then determined. Based on this histogram the following SD indices were calculated:

– wpsum02 = relative portion of words consisting only of the symbols “0” and “2”, a measure for decreased HRV,
– wpsum13 = relative portion of words consisting only of the symbols “1” and “3”, a measure for increased HRV,
– pW000 to pW333 = probability of occurrence of each single word type (000, 001, …, 333) within NN interval time series,
– pTH1 to pTH20 = number of words with a probability higher than a threshold level pTH (1–20%).

In a further SD algorithm, symbol strings using the alphabet *A* = {0, 1} were generated. Here the symbol “0” represents differences between two successive NN-intervals lower than a special limit (e.g., 5 ms), whereas symbol “1” indicates differences that are equal or higher to this selected limit. From the symbol strings, words consisting of 6 successive equal symbols were achieved to detect epochs of low or high variability. The use of 2 symbols and of a word length of 6 symbols leads to 64 different word pattern (2^6^). 30 min ECG recordings with a mean heart rate of 60bpm contain approximately 1800 NN intervals, resulting in about 28 words per word pattern (in case of a uniform distribution). According to Voss et al. (1996), a heuristic basis of 20 as the averaged minimal number of words per word pattern is required. A lower number of words per word pattern would reduce the accuracy of the word distribution estimation. The most interesting word pattern consists of the symbol sequence “111111” quantifying high variability epochs (phvar) and of the sequence “000000” quantifying low variability (plvar) within the NN interval time series. Additionally, we calculated the following SD indices:

– phvar5 = portion of high-variability patterns within the NN interval time series (>5 ms),
– plvar5 = portion of low-variability patterns within the NN interval time series (<5 ms).

***Segmented short-term symbolic dynamics***. The segmented short-term SD (SSD applied only for 24 h ECG analysis) represents a quiet new short-term SD approach that was introduced to describe nonlinear aspects within long-term NN interval time series in an enhanced way (Voss et al., 2010b). SSD is based on the classical SD analysis approach and corresponds to SD transformation rules (Voss et al., 1996). In contrast to the classical SD, SSD was adapted for calculating long-term time series by applying a 24-h segmentation algorithm. Along these lines, 24 h NN interval time series were segmented into time windows of 30 min duration by applying an overlap of 29 min (shift = 1 min). For each window (s = 1… S, S as the number of segments) the following classical SD indices were estimated as:

– pW000*s* to pW333*s* = probability of occurrence of each word type (000, 001, …, 333) within 30 min NN interval time series,
– pTH1*s* to pTH20*s* = number of words with a probability higher than a threshold pTH (1–20%).

Subsequently, the mean values (m_pW000 to m_pW333) and standard deviations (s_pW000 to s_pW333) of the indices pW000*s* to pW333*s* and pTH1*s* to pTH20*s* were calculated.

The Shannon entropy, calculated from the distribution of each single word type over all windows, was estimated to be a suitable measure to quantify the dynamic behavior and complexity of word type occurrences within the windowed time series. For example, the Shannon entropy [bit] of the word type “111”, applying the probability of occurrence pW111_*i*_ of each bin (*i* = 1… nob; nob is the number of bins determined via Sturges criterion: nob = 1 + 3.32xlog(S)) is shown by:

$$\text{Shannon}\_\text{pW1}11\left[\text{bit}\right]=-\sum_{i=1}^{\text{nob}}\text{pW}111_{i}\;\times \;\log_{2}\text{pW}111_{i}.$$

When calculating SSD, only one NN interval segment (window) of 30 min duration can be analyzed in case of investigation of 30 min NN interval time series, whereby SSD is then equal to SD.

***Short-term symbolic dynamics***. Porta et al. (2001) introduced a modified procedure of SD: the short-term SD (STSD). Here the length of the RR-intervals was limited to 300 NN intervals. Firstly, STSD transforms the NN interval time series into symbol strings using the alphabet A = {0, 1, 2, 3, 4, 5}. To this aim, the full range of the sequences is uniformly spread out on 6 levels (0–5). Subsequently, word patterns of length three were constructed using the symbol strings, resulting in 6^3^ = 216 possible word types. Finally, all word patterns were grouped without a loss into pattern families, and the probability of occurrence for each pattern family was calculated:

– ST_0V = portion of word patterns with no variation (three successive symbols are equal, e.g., “111” or “222”),
– ST_1V = portion of word patterns with 1 variation (e.g., “355” or “212”),
– ST_2V = portion of word patterns with 2 variations (e.g., “123” or “534”).

Furthermore, the indices of some additional STSD pattern families were calculated (Heitmann et al., 2011):

– ST_ASC = portion of word patterns where three successive symbols form an ascending ramp, e.g., “234” or “012”),
– ST_DESC = portion of word patterns where three successive symbols form a descending ramp, e.g., “432” or “210”),
– ST_PEAK = portion of word patterns where the second symbol is larger than the other two symbols, forming a peak, e.g., “121” or “243”),
– ST_VAL = portion of word patterns where the second symbol is smaller than the other two symbols, forming a valley, e.g., “212” or “312”),
– ST_PLATEAU = portion of word patterns where two successive symbols are equal (Plateau) and the remaining one symbol differs, e.g., “355” or “221”).

In this study, STSD was adapted for calculating 24 h and 30 min time series by applying a segmentation algorithm that segments the investigated NN time series into non-overlapping windows of 300 NN interval duration. The above-introduced STSD indices were estimated for each window. Afterwards, the mean value (m_) and standard deviation (s_) of each STSD index were calculated over all windows, e.g., m_ST_1V and s_ST_1V.

***Standard deviation coded symbolic dynamics***. Another SD approach was introduced by Mrowka et al. (1997)—the standard deviation coded SD (SDSD) - where the symbol transformation (A = {0, 1, 2, 3, 4}) of the NN interval time series was performed by applying a sliding NN interval window j of length M that is shifted (τ) for the entire NN interval time series of length L. In each window, the number of consecutive NN interval differences that have decreased compared to the a-scaled standard deviation of the current window will be determined which correspond to the symbol Sj of alphabet A. During this study we used a = 1, τ =1, M = 5, and L = 24 h or 30 min. We calculated that:

– tau1_p001 = the number of symbol types with a probability of occurrence higher than 1%.

#### Detrended fluctuation analysis

To investigate the self-affinity of NN time series fluctuations over multiple time scales, the detrended fluctuation analysis (DFA), based on a modified random walk analysis, was introduced and applied to physiologic time series by Peng et al. (1995). DFA quantifies the presence or absence of fractal correlation properties in noisy and non-stationary time series. Therefore, the NN interval time series were integrated *y*(*k*)(*k* = 1, …, *N*, *N* as the length of the NN time series) and divided into equal and non-overlapping segments of length *n*. For each segment, the local trend *y*_*n*_(*k*) was determined by least-squares fitting and subtracted from *y*(*k*). Root-mean-square fluctuation values *F*(*n*) were then calculated. Finally, two scaling exponents were calculated as the slope of the double-log plot of *F*(*n*) against *n*.

For short-term fractal scaling, properties over a range of 4–16 beats (α1) and for long-term fractal scaling, properties over a range of 16–64 beats (α2) were calculated.

#### Segmented Poincaré plot analysis

Voss et al. (2010a) introduced the segmented Poincaré plot analysis (SPPA), based on the traditional Poincaré plot analysis (PPA) (Kamen and Tonkin, 1995), which is a nonlinear quantitative technique of phase-space characterization. In contrast to PPA, SPPA avoids linear correlation and quantifies nonlinear features of NN time series.

Here the 45-degree rotated cloud of points is segmented into 12 × 12 equal rectangles whose size depends on SD1 (height) and SD2 (width) of the Poincaré plot. Several SPPA indices representing the percentage of points found in each column and row compared to the rate of all points were estimated based on a 12 × 12-probability matrix:

– SPPA_r_i = single probability of each row with *i* = 1–12,
– SPPA_c_j = single probability of each column with *j* = 1–12,
– SPPA_entropy = Shannon entropy of the 12 × 12 probability matrix [bit].

### Patients

In the MUSIC2 study (see section Data Acquisition and Pre-processing), 256 patients with symptomatic IHF were enrolled during the first part (of the MUSIC2 study). The MUSIC2 study was designed to assess risk factors for SCD in HF patients with mild to moderate HF. HF diagnosis was confirmed by experienced cardiologists via radiography (findings of pulmonary congestion) and/or echocardiography (abnormal left ventricular filling pattern and left ventricular hypertrophy), also via short- and long-term ECG and/or stress ECG. Heart failure symptoms corresponding to NYHA class II or III, associated with echocardiographic signs of systolic and/or diastolic dysfunction and sinus rhythm, were defined as inclusion criteria. Exclusion criteria were recent acute coronary syndrome or severe valvular disease, severe pulmonary, hepatic, or renal disease or other concomitant no cardiovascular disease expected to reduce life expectancy to less than 3 years. Furthermore, in this study only patients with a time lag of more than 3 months after their last hospitalization due to HF decompensation were enrolled. In addition, patients with a percentage of ectopic beats or artefacts in the NN time series more than 10% were excluded from analysis to minimize filtering influences on the final analysis results. All IHF patients (♂ = 210, ♀ = 46) received optimal medical treatment with ACE inhibitors (73%), beta-blockers (75%) and diuretics (57%) in accordance with institutional guidelines.

Clinical risk factors were included in this study:

– LVEF [%] = left ventricular ejection fraction,
– NYHA = New York Heart Association functional class,
– BMI [kg/m^2^] = body mass index,
– LVDD [mm] = left ventricular end-diastolic diameter,
– LVSD [mm] = left ventricular systolic diameter
– NT-proBNP [pmol/l] = plasma concentration of N-terminal pro-brain natriuretic peptide.

After a 2-year follow-up, all IHF patients were split into two subgroups according to survival: survivors (low risk group—IHF_LR_: *n* = 221, ♂ = 180, ♀ = 41; median age: 63 [56–71] years), and non-survivors (high risk group—IHF_HR_: *n* = 35, ♂ = 30, ♀ = 5; median age: 68 [61–72] years) including patients that died due to a cardiac event. Age and gender distribution of both subgroups did not significantly differ (age: *p* = 0.10, gender: *p* = 0.64). In the IHF_HR_ subgroup, 17 IHF patients who suffered from SCD were included. The investigation conformed to the recommendations of the Declaration of Helsinki. The ethical committee of the respective institutions approved the study protocol; all patients gave their written informed consent before study participation.

### Statistics

Univariate statistical analyses using the nonparametric exact two-tailed Mann-Whitney *U*-test (SPPS 21.0) was performed to non-normally distributed indices (significant Kolmogorov-Smirnov test) to evaluate differences between IHF_LR_ and IHF_HR_ for these series: the 24 h NN time series, the first 30 min segments of NN interval time series, the 30 min most stationary day segments of NN interval time series, and the 30 min most stationary night segments of NN interval time series. Significances were based on values of *p* < 0.05. Due to multiple testing, the univariate significance level was adjusted to *p* < 0.01. In addition and based on descriptive statistics, mean values, standard deviations, medians, and lower and upper quartiles were calculated.

Multivariate analysis based on discriminant analysis in combination with receiver operator characteristic (ROC) curves was applied only to uncorrelated (Pearson correlation coefficient) univariate significant indices. The sensitivity (SENS), specificity (SPEC), area under the ROC curve (AUC) and positive predictive accuracy (PPA) were determined for different parameter sets, each consisting of five indices:

– Clinical indices sets,
– Non-clinical indices sets and
– Mixed sets of both clinical and non-clinical indices: mixed set 1 − a parameter set consisting of three clinical indices and two non-clinical indices; mixed set 2 − a parameter set consisting of two clinical indices and three non-clinical indices.

## Results

### Univariate analysis

According to the univariate discrimination between IHF patients at low risk and high risk respectively, both clinical and non-clinical indices could prove their ability to differentiate between these two patient groups. Table 1 presents the results of the clinical indices and Tables 2–5 show the findings of the non-clinical indices.

**Table 1:** **Clinical indices: univariate statistical analysis results (*U*-test) to discriminate between patients with ischemic heart failure at low risk and at high risk (IHF_LR_ and IHF_HR_)**.

**Clinical index**	**IHF_LR_**	**IHF_HR_**	***p***
BMI [kg/m^2^]^b^	29 [26–32]	27 [24–28]	0.0019^#^
LVDD [mm]^b^	61 [56–67]	65 [60–67]	0.1125^ns^
LVEF [%]^b^	35 [26–40]	30 [25–35]	0.0211^*^
LVSD [mm]^b^	49 [41–57]	53 [51–58]	0.0233^*^
NT-ProBNP [pmol/l]^b^	70 [34–153]	184 [108–583]	2.7 × 10^−6^^†^
NYHA (class: II/III)^a^	189/32 (85.5%/14.5%)	19/16 (54.3%/45.7%)	0.0001^†^

a *Number of patients (percentage)*,

b *median [lower (0.52)—upper (0.75) quartile]*,

p - univariate significance (

ns *not significant*,

* *p < 0.05*,

# *p < 0.01*,

† *p < 0.001)*.

**Table 2:** **Analysis of 24 h Holter ECGs: univariate statistical analysis results (*U*-test) to discriminate between patients with ischemic heart failure at low risk and at high risk (IHF_LR_ and IHF_HR_)**.

**Method**	**Index**	**IHF_LR__24 h**	**IHF_HR__24 h**	**p_24 h**
HRV	meanNN [ms]^a^	859 [773–951]	816 [730–877]	0.0431^*^
	sdNN [ms]^a^	103 [80–130]	92 [60–127]	0.1184^ns^
	rmssd [ms]^a^	23.46 [17.13–32.74]	24.19 [10.9–34.68]	0.6774^ns^
	LF/HF^a^	2.52 [1.71–3.87]	1.74 [1.20–2.63]	0.0022^#^
	VLF/P^a^	0.11 [0.07–0.15]	0.08 [0.05–0.14]	0.0864^ns^
SD	wpsum13^a^	0.61 [0.51–0.68]	0.54 [0.43–0.67]	0.0362^*^
	pW231^a^	4.5x10^−5^ [1.2 × 10^−5^–1.5 × 10^−4^]	7.1 × 10^−5^ [0–3.9 × 10^−4^]	0.3441^ns^
	pW333^a^	0.30 [0.26–0.34]	0.25 [0.22–0.33]	0.0216^*^
	plvar5^a^	1.7 × 10^−4^ [4.2 × 10^−5^–7.1 × 10^−4^]	3.6 × 10^−4^ [4.1 × 10^−5^–4.4 × 10^−3^]	0.0512^ns^
STSD	m_ST_PEAK^a^	0.09 [0.07–0.11]	0.10 [0.08–0.13]	0.0130^*^
	m_ST_VAL^a^	0.10 [0.08–0.12]	0.11 [0.09–0.14]	0.0135^*^
	s_ST_PLATEAU^a^	0.09 [0.08–0.10]	0.08 [0.07–0.09]	0.0099^#^
SDSD	tau1_p001^b^	4.86 ± 0.35	4.60 ± 0.50	0.0008^†^
DFA	α1^a^	1.20 [1.06–1.33]	1.04 [0.90–1.25]	0.0005^†^
SPPA	SPPA_entropy [bit]^a^	3.93 [3.80–4.01]	3.90 [3.77–4.00]	0.6194^ns^
	SPPA_r_5^a^	7.86 [5.64–9.89]	7.90 [5.80–9.32]	0.8640^ns^
	SPPA_r_10^a^	0.40 [0.28–0.51]	0.39 [0.29–0.56]	0.6246^ns^
SSD	s_pW111^a^	0.10 [0.09–0.12]	0.09 [0.07–0.11]	0.0073^#^
	Shannon_pW233 [bit]^a^	2.73 [2.54–2.90]	2.60 [2.13–2.77]	0.0023^#^
	Shannon_pW332 [bit]^a^	2.74 [2.54–2.90]	2.57 [2.17–2.74]	0.0004^†^
	Shannon_pW333 [bit]^a^	2.80 [2.61–2.93]	2.68 [2.21–2.82]	0.0025^#^
	m_pTH5^a^	4.02 [3.74–4.43]	3.72 [3.46–4.11]	0.0073^#^
	m_pTH6^a^	3.51 [3.25–3.79]	3.32 [3.00–3.55]	0.0032^#^
	m_pTH7^a^	3.17 [2.96–3.41]	3.02 [2.67–3.22]	0.0054^#^

Methods: HRV, standard heart rate variability analysis (time- and frequency domain), SD, classical symbolic dynamics; STSD, short-term symbolic dynamics; SDSD, standard deviation coded symbolic dynamics; DFA, detrended fluctuation analysis; SPPA, segmented Poincaré plot analysis; SSD, segmented short-term symbolic dynamics;

other abbreviations:

a *median [lower (0.52)—upper (0.75) quartile]*,

b *mean value ± standard deviation*,

p—univariate significance (

ns *not significant*,

* *p < 0.05*,

# *p < 0.01*,

† *p < 0.001)*.

**Table 3:** **Analysis of first 30 min ECG segments: univariate statistical analysis results (*U*-test) to discriminate between patients with ischemic heart failure at low risk and at high risk (IHF_LR_ and IHF_HR_)**.

**Method**	**Index**	**IHF_LR__30 min**	**IHF_HR__30 min**	**p_30 min**
HRV	meanNN [ms]^a^	804 [703–918]	716 [671–825]	0.0165^*^
	sdNN [ms]^a^	62.01 [41.51–85.98]	51.18 [28.01–79.65]	0.1046^ns^
	rmssd [ms]^a^	18.50 [13.34–27.32]	21.04 [11.13–27.45]	0.7504^ns^
	LF/HF^a^	2.93 [1.71–5.33]	1.88 [1.08–3.63]	0.0032^#^
	VLF/P^a^	0.24 [0.13–0.39]	0.20 [0.08–0.37]	0.2355^ns^
SD	wpsum13^a^	0.39 [0.24–0.58]	0.30 [0.17–0.52]	0.1416^ns^
	pW231^a^	0 [0–0]	0 [0–0]	0.3311^ns^
	pW333^a^	0.19 [0.13–0.28]	0.18 [0.09–0.24]	0.1040^ns^
	plvar5^a^	3.6 × 10^−3^ [5.4 × 10^−4^–2.0 × 10^−2^]	7.5 × 10^−3^ [1.9 × 10^−3^–4.3 × 10^−2^]	0.0673^ns^
STSD	m_ST_PEAK^a^	0.09 [0.07–0.12]	0.11 [0.08–0.13]	0.0074^#^
	m_ST_VAL^a^	0.10 [0.08–0.12]	0.12 [0.09–0.14]	0.0072^#^
	s_ST_PLATEAU^a^	0.07 [0.05–0.09]	0.06 [0.05–0.08]	0.7281^ns^
SDSD	tau1_p001^b^	4.81 ± 0.40	4.51 ± 0.51	0.0004^†^
DFA	α1^a^	1.23 [1.03–1.36]	1.01 [0.85–1.26]	0.0002^†^
SPPA	SPPA_entropy [bit]^a^	3.96 [3.82–4.06]	3.86 [3.68–3.98]	0.0024^#^
	SPPA_r_5^a^	9.79 [7.19–12.25]	7.95 [6.32–10.47]	0.0100^#^
	SPPA_r_10^a^	0.30 [0.18–0.49]	0.35 [0.23–0.58]	0.1553^ns^

Methods: HRV, standard heart rate variability analysis (time- and frequency domain), SD, classical symbolic dynamics; STSD, short-term symbolic dynamics; SDSD, standard deviation coded symbolic dynamics; DFA, detrended fluctuation analysis; SPPA, segmented Poincaré plot analysis;

other abbreviations:

a *median [lower (0.52)–upper (0.75) quartile]*,

b *mean value ± standard deviation*,

p—univariate significance (

ns *not significant*,

* *p < 0.05*,

# *p < 0.01*,

† *p < 0.001)*.

**Table 4:** **Analysis of 30 min most stationary beat-to-beat interval segments of the day phase—univariate statistical analysis results (*U*–test) to discriminate between patients with ischemic heart failure at low risk and at high risk (IHF_LR_ and IHF_HR_)**.

**Method**	**Index**	**IHF_LR__day**	**IHF_HR__day**	**p_day**
HRV	meanNN [ms]^a^	807 [730–902]	765 [703–881]	0.0964^ns^
	sdNN [ms]^a^	33.64 [23.42–52.90]	32.43 [19.32–48.43]	0.1862^ns^
	rmssd [ms]^a^	15.93 [11.15–24.33]	13.79 [8.96–29.40]	0.4079^ns^
	LF/HF^a^	3.11 [1.74–4.99]	1.97 [0.77–3.05]	0.0007^†^
	VLF/P^a^	0.47 [0.34–0.57]	0.36 [0.16–0.48]	0.0026^#^
SD	wpsum13^a^	0.11 [0.04–0.24]	0.09 [0.02–0.23]	0.2403^ns^
	pW231^a^	0 [0–0]	0 [0–4.1x10^−4^]	0.3001^ns^
	pW333^a^	0.06 [0.03–0.13]	0.05 [0.01–0.12]	0.1731^ns^
	plvar5^a^	0 [0–4.0x10^−4^]	0 [0–1.7x10^−3^]	0.0160^*^
STSD	m_ST_PEAK^a^	0.09 [0.07–0.13]	0.11 [0.08–0.13]	0.1226^ns^
	m_ST_VAL^a^	0.10 [0.08–0.14]	0.11 [0.08–0.14]	0.3533^ns^
	s_ST_PLATEAU^a^	0.06 [0.04–0.08]	0.07 [0.04–0.08]	0.5437^ns^
SDSD	tau1_p001^b^	4.75 ± 0.44	4.66 ± 0.48	0.3036^ns^
DFA	α1^a^	1.21 [1.02–1.35]	1.04 [0.85–1.24]	0.0070^#^
SPPA	SPPA_entropy [bit]^a^	3.98 [3.81–4.09]	3.97 [3.79–4.07]	0.4538^ns^
	SPPA_r_5^a^	9.96 [7.51–12.97]	8.94 [7.23–12.11]	0.3444^ns^
	SPPA_r_10^a^	0.24 [0.14–0.40]	0.39 [0.25–0.62]	0.0018^#^

Methods: HRV, standard heart rate variability analysis (time- and frequency domain); SD, classical symbolic dynamics; STSD, short-term symbolic dynamics; SDSD, standard deviation coded symbolic dynamics; DFA, detrended fluctuation analysis; SPPA, segmented Poincaré plot analysis;

other abbreviations:

a *median [lower (0.52)–upper (0.75) quartile]*,

b *mean value ± standard deviation*,

p—univariate significance (

ns *not significant*,

* *p < 0.05*,

# *p < 0.01*,

† *p < 0.001)*.

**Table 5:** **Analysis of 30 min most stationary beat-to-beat interval segments of the night phase- univariate statistical analysis results (*U*–test) to discriminate between patients with ischemic heart failure at low risk and at high risk (IHF_LR_ and IHF_HR_)**.

**Method**	**Index**	**IHF_LR__night**	**IHF_HR__night**	**p_night**
HRV	meanNN [ms]^a^	900 [795–1009]	874 [759–928]	0.1920^ns^
	sdNN [ms]^a^	34.86 [22.61–54.35]	29.13 [14.75–44.73]	0.0188^*^
	rmssd [ms]^a^	20.04 [13.13–27.62]	19.16 [7.75–32.27]	0.3701^ns^
	LF/HF^a^	2.29 [1.21–3.93]	1.18 [0.65–3.60]	0.0186^*^
	VLF/P^a^	0.44 [0.32–0.57]	0.38 [0.26–0.51]	0.0444^*^
SD	wpsum13^a^	0.07 [0.02–0.16]	0.04 [0–0.10]	0.0056^#^
	pW231^a^	0 [0–0]	0 [0–4.8 × 10^−4^]	0.0023^#^
	pW333^a^	0.05 [0.01–0.09]	0.02 [0–0.07]	0.0014^#^
	plvar5^a^	0 [0–0]	0 [0–1.4 × 10^−3^]	0.0052^#^
STSD	m_ST_PEAK^a^	0.09 [0.07–0.13]	0.11 [0.09–0.15]	0.0072^#^
	m_ST_VAL^a^	0.10 [0.07–0.14]	0.12 [0.10–0.17]	0.0035^#^
	s_ST_PLATEAU^a^	0.06 [0.04–0.08]	0.06 [0.04–0.07]	0.8238^ns^
SDSD	tau1_p001^b^	4.73 ± 0.45	4.49 ± 0.51	0.0055^#^
DFA	α1^a^	1.20 [1.02–1.35]	1.03 [0.67–1.26]	0.0046^#^
SPPA	SPPA_entropy [bit]^a^	3.97 [3.80–4.05]	3.92 [3.76–4.09]	0.5769^ns^
	SPPA_r_5^a^	11.39 [8.52–14.12]	11.14 [6.81–13.82]	0.4960^ns^
	SPPA_r_10^a^	0.17 [0.07–0.33]	0.13 [0.08–0.27]	0.4117^ns^

Methods: HRV, standard heart rate variability analysis (time- and frequency domain); SD, classical symbolic dynamics; STSD, short-term symbolic dynamics; SDSD, standard deviation coded symbolic dynamics; DFA, detrended fluctuation analysis; SPPA, segmented Poincaré plot analysis;

other abbreviations:

a *median [lower (0.52)—upper (0.75) quartile]*,

b *mean value ± standard deviation*,

p—univariate significance (

ns *not significant*,

* *p < 0.05*,

# *p < 0.01)*.

#### Clinical indices

**Figure 1:**
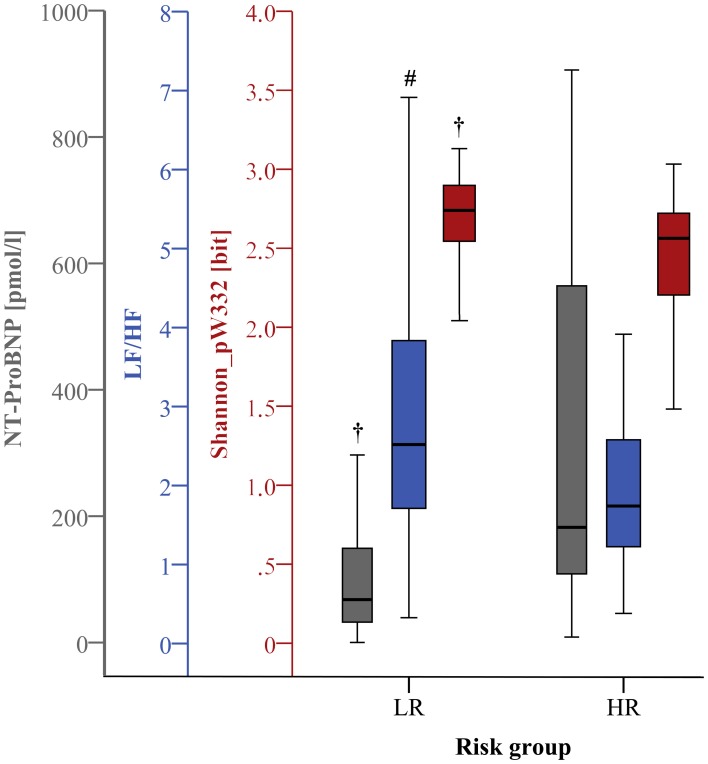
**Boxplots of the most significant univariate clinical, linear, and nonlinear indices (24 h) discriminating low (LR) and high (HR) risk groups (^#^*p* < 0.01; ^†^*p* < 0.001)**.

As shown in Table 1, the BMI of the high-risk IHF group was considerably lower (*p* < 0.01) than the BMI of the low-risk IHF group. From echocardiographic indices, LVEF and LVSD revealed slightly significant (*p* < 0.05) differences between IHF_LR_ and IHF_HR_. LVDD was similar when comparing both groups. The IHF_HR_ group was characterized by a decreased LVEF and an increased LVSD when compared to the IHF_LR_ group. Both the biomarker NT-ProBNP (Figure 1) and the subjective NYHA index were dramatically higher (*p* < 0.001) in patients at high risk, than in patients at low risk.

#### Non-clinical indices

Tables 2–5 illustrate the statistical results for non-clinical indices, differentiating between IHF_LR_ and IHF_HR_ and investigating these series: 24 h NN time series (Table 2), the first 30 min segments of NN interval time series (Table 3), the 30 min most stationary day segments of NN interval time series (Table 4) and the 30 min most stationary night segments of NN interval time series (Table 5). In summary, most of the indices decreased at least in trend within the IHF_HR_ group when compared to the IHF_LR_ group, independent of the considered length of the NN interval time series and the day/night phase. With regard to the investigated time length and day/night phase, only indices LF/HF from the frequency domain and α1 from the DFA revealed comparably significant differences between both risk groups (*p* < 0.01). In contrast, all other indices showed differences in terms of significance. The number of significant indices (*p* < 0.05) was highest when using 24 h NN interval time series (*n* = 16, of which seven indices were from the SSD method which was not applied for 30 min analysis), followed by 30 min most stationary night phase (*n* = 11), first 30 min of 24 h NN interval time series (*n* = 8), and 30 min most stationary day phase (*n* = 5).

**Figure 2:**
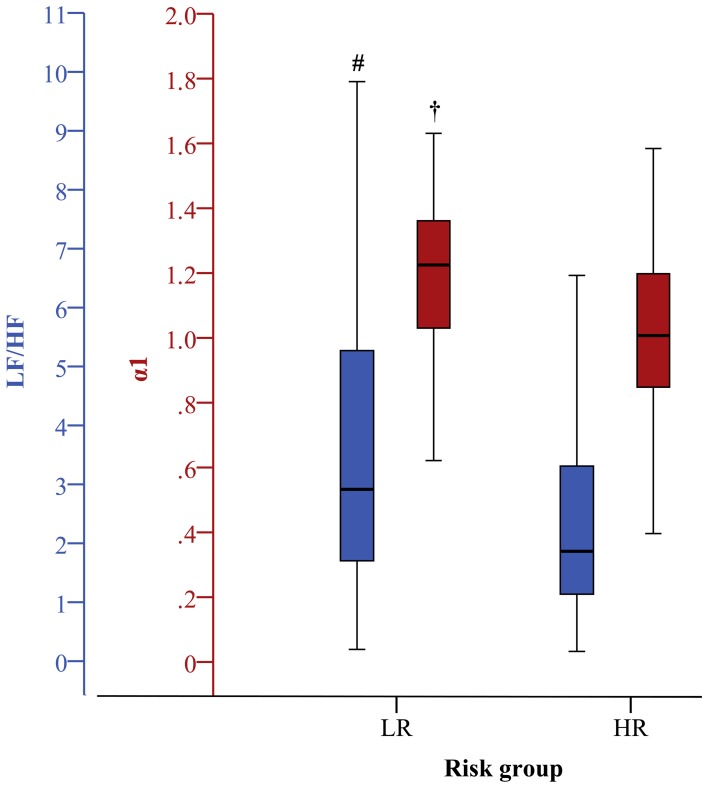
**Boxplots of the most significant univariate linear and nonlinear indices (first 30 min) discriminating low (LR) and high (HR) risk groups (^#^*p* < 0.01; ^†^*p* < 0.001)**. The clinical index is the same as in Figure 1.

**Figure 3:**
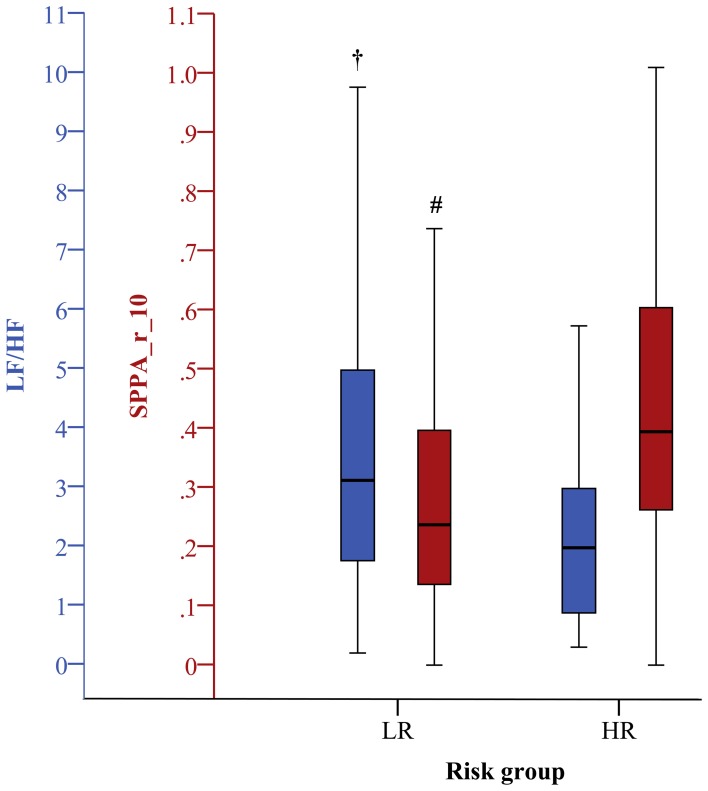
**Boxplots of the most significant univariate clinical, linear, and nonlinear indices (30 min day phase) discriminating low (LR) and high (HR) risk groups (^#^*p* < 0.01; ^†^*p* < 0.001)**.

**Figure 4:**
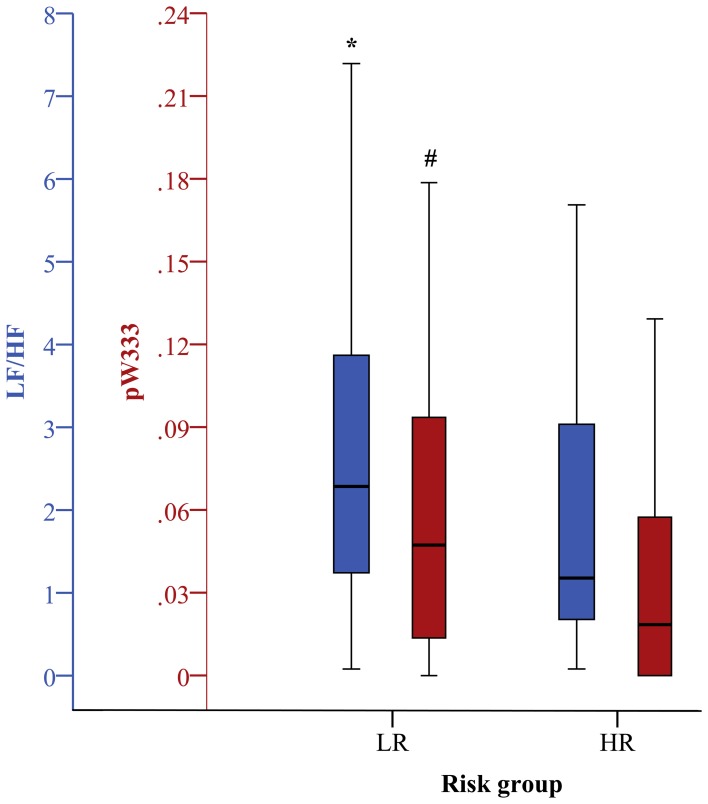
Boxplots of the most significant univariate clinical, linear, and nonlinear indices (30 min night phase) discriminating low (LR) and high (HR) risk groups (^*^p < 0.05; ^#^*p* < 0.01).

***Linear time and frequency domain HRV indices***. Based on the TD of traditional linear HRV analysis (Task Force, 1996), the meanNN significantly decreased (*p* < 0.05) in IHF_HR_ patients when compared to IHF_LR_ patients considering 24 h NN interval time series and their first 30 min segments. Due to the selection criteria of the most-stationary NN interval segments, the decrease of meanNN within IHF_HR_ was not significant, or at least in trend different after analysing the 30 min day and night segments, respectively. With regard to the 30 min night segment, sdNN was significantly reduced (*p* < 0.05) in the IHF_HR_ patients group. For all other analyzed time segments, sdNN was similar in both patient groups. From frequency domain, LF/HF, which describes the sympathovagal balance, diminished (*p* < 0.05) in the IHF_HR_ group when compared to the IHF_LR_ group (Figures 1–4); this was independent of the investigated NN interval duration (24 h or any 30 min). The most considerable decrease (*p* < 0.001) of LF/HF in IHF_HR_ was noticed by HRV analysis of 30 min most stationary day NN interval segments. With regard to IHF_LR_ patients, analysis of the 30 min day and night segments respectively revealed a significantly reduced VLF/P index (*p* < 0.05) in IHF_HR_ patients.

**Figure 5:**
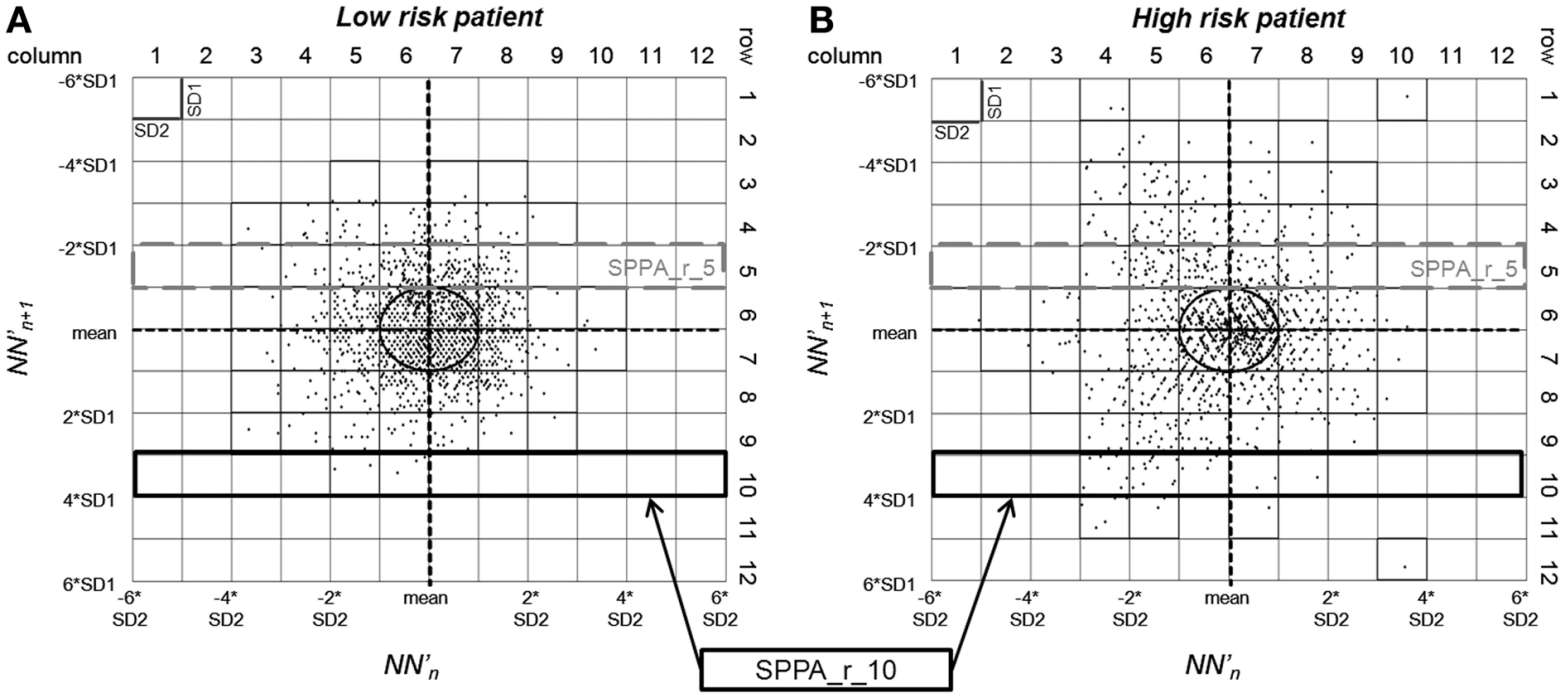
**SPPA plot with marked indices SPPA_r_5 and SPPA_r_10 of two patients (A): low risk, (B): high risk**.

***Nonlinear HRV indices***. From nonlinear domain, particularly indices from symbolic dynamics considerably proved their ability to differentiate between IHF patients at low and high risk of cardiac death. Considering the classical symbolic dynamics, the index wpsum13 and the occurrence probability of word type “333” (pW333) significantly decreased in IHF_HR_ patients when performing 24 h analysis (*p* < 0.05). This was particularly the case for the analysis of 30 min night (Figure 4) time series (*p* < 0.01). During the most stationary night phase, word type “231” occurred more frequently (pW231, *p* < 0.01) in the IHF_HR_ patient group related to IHF_LR_. The portion of low-variability patterns (differences <5 ms) in NN interval time series (plvar5) for 30 min day- and night phases were significantly larger (*p* < 0.05) in IHF_HR_ patients than in IHF_LR_ patients. From STSD, the relative number of word patterns consisting of three symbols with an increased (m_ST_PEAK) or a decreased (m_ST_VAL) middle symbol was higher in the IHF_HR_ patient group than in the IHF_LR_ patient group. This was particularly the case for analysis of 30 min NN interval time series (first 30 min and night phase, *p* < 0.01), but also for results of the 24 h HRV analysis (*p* < 0.05). The standard deviation of word patterns forming a plateau was clearly decreased (*p* < 0.01) in IHF_HR_, but only when investigating the 24 h time series. In contrast to IHF_LR_, tau1_p001 from the SDSD method noticeably decreased in the IHF_HR_ group, particularly considering 24 h and first 30 min NN interval time series (*p* < 0.001), but also when considering the 30 min night NN interval segments (*p* < 0.01). As previously explained in the method section, the segmented mode of classical symbolic dynamics SSD was applied only for the analysis of 24 h NN interval time series. From SSD, seven indices (s_pW111, Shannon_pW233, Shannon_pW332 (Figure 1), Shannon_pW333, m_pTH5, m_pTH6, m_pTH7) revealed their ability to differentiate considerably (at least *p* < 0.01) when comparing IHF_HR_ and IHF_LR_ patients. All SSD indices were decreased in the high-risk patient group when compared to low-risk patients. Beside the indices from symbolic dynamics, indices from DFA and SPPA could also demonstrate their capacity to differentiate between low- and high-risk IHF patients, at least in part. Particularly, independent of the considered duration of time series and the different 30 min NN interval segments (first 30 min, day and night), α1 from DFA was highly reduced (24 h/first 30 min: *p* < 0.001 and day/night: *p* < 0.01) in the IHF_HR_ patients group compared to the IHF_LR_ group (Figure 2). From SPPA, the indices SPPA_entropy and SPPA_r_5 were significantly decreased (*p* < 0.01) in the IHF_HR_ group, but only if investigating the first 30 min NN interval segments. Furthermore, regarding to IHF_HR_ patients, SPPA_r_10 was increased (*p* < 0.01) only during the 30 min most stationary day phase (Figure 3). In Figure 5 examples of index SPPA_r_10 for LR and HR are presented.

### Multivariate analysis

According to the multivariate discrimination between IHF patients at low- and high risk, optimal clinical indices sets, optimal non-clinical indices sets and optimal mixed sets of both clinical and non-clinical indices, each consisting of five indices, were determined. Table 6 presents the discriminating results with regard to the analysis of 24 h NN interval time series and their first 30 min NN segments, as well as the results of the 30 min most stationary day and night segments.

**Table 6:**
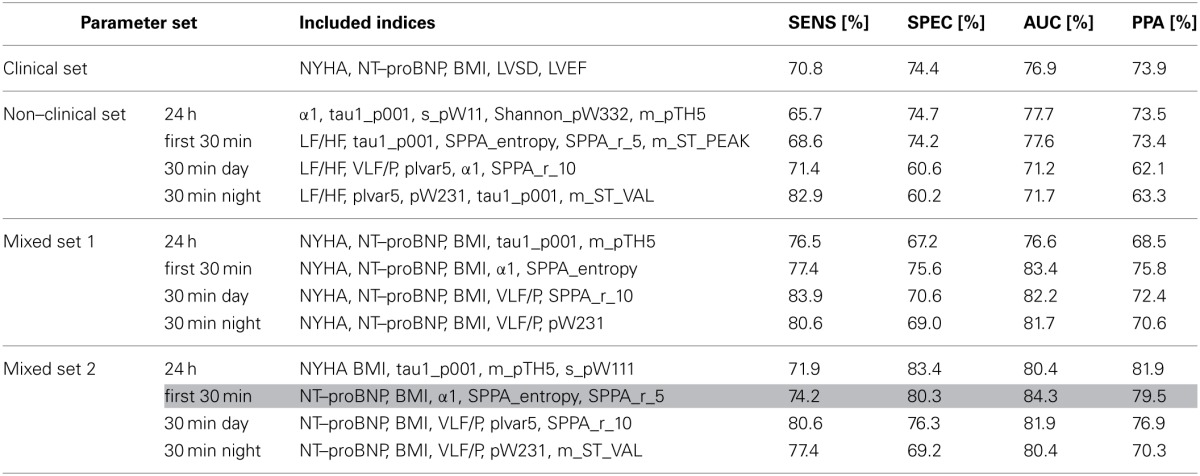
**Multivariate classification results (discriminant analysis) of a clinical parameter set and 3 × 4 different optimal parameter sets (one non–clinical set, two mixed sets estimated for each 24 h, first 30 min, and 30 min most stationary day and night beat–to–beat time series) consisting each of five indices**.

Abbreviations *SENS* *sensitivity* *SPEC* *specificity* *AUC* *area under the receiver operating characteristic curve* *PPA* *positive predictive accuracy* *gray-marked row* *parameter set with the highest values for AUC and PPA* *Mixed set 1* *parameter set consisting of three clinical indices and two non-clinical indices* *Mixed set 2* *parameter set consisting of two clinical indices and three non-clinical indices*.

The optimal non-clinical sets obtained from the analysis of 24 h (α1, tau1_p001, s_pW111, Shannon_pW332, m_pTH5) and first 30 min (LF/HF, tau1_p001, SPPA_entropy, SPPA_r_5, m_ST_PEAK) NN interval time series revealed a similar AUC and PPA when compared to the optimal clinical parameter set (NYHA, NT-ProBNP, BMI, LVSD, LVEF). The AUC between IHF_HR_ and IHF_LR_ was noticeably lower for optimal non-clinical parameter sets of day (LF/HF, VLF/P, plvar5, α1, SPPA_r_10) and night (LF/HF, plvar5, pW231, tau1_p001, m_ST_VAL) NN interval segments than the AUC of the clinical parameter set or the non-clinical parameter sets from 24 h and first 30 min HRV analysis. In general, the mixture of both clinical and non-clinical indices leads to an increased AUC when compared to a purely clinical or non-clinical parameter set, independent of the considered NN interval segments (the first 30 min, day or night) or their durations (24 h or 30 min). The optimal mixed parameter set regarding 24 h HRV analysis consists of two clinical indices (NYHA and BMI) and three non-clinical indices (tau1_p001 from SDSD, m_pTH5 and s_pW111 from SSD). With respect to the analysis of the first 30min NN interval segments, the optimal parameter set consists of two clinical indices (NT-ProBNP and BMI) and three indices from two analysis methods (α1 from DFA, SPPA_entropy and SPPA_r_5 from SPPA). Concerning the analysis of the 30 min most stationary day and night NN interval segments, optimal AUC values were achieved by mixing three clinical indices (NYHA, NT-ProBNP, BMI) and two non-clinical indices (day - VLF/P from frequency domain and SPPA_r_10 from SPPA; night - VLF/P, and pW231 from SD). The best AUC value (84.3%) was obtained using the optimal mixed parameter set from the first 30 min HRV analysis. The AUC value ranges from the above-mentioned optimal mixed parameter sets are comparable (80.4–84.3%).

## Discussion

In this study, we investigated whether 30 min short-term HRV analysis is sufficient for comparable risk stratification in IHF patients in comparison to 24 h HRV analysis. In a previous study (Voss et al., 2010b), an optimal mixed parameter set consisting of nonlinear indices from long-term (24 h) HRV analysis and clinical indices achieved the highest discrimination power for detecting IHF patients at high risk of cardiac death, resulting in an enhanced risk stratification in IHF patients. Therefore, to investigate if HRV analysis of 30 min ECGs leads to a similar level of discrimination power when compared to the analysis of 24 h ECGs, we also included available clinical indices.

### Univariate analysis

#### Clinical indices

The finding of a lowered clinical BMI in patients from the high risk group was also reported in several studies (Pocock et al., 2006; Goode et al., 2008; Cai et al., 2012), describing the BMI as an independent predictor of cardiac death in heart failure patients. Both increased values of LVDD and LVSD in heart failure patients were found to be independent risk predictors (Nolan et al., 1998; Bruch et al., 2005) and were also enlarged in IHF patients at high risk of cardiac death in our study. According to (Pocock et al., 2006), strong predictors for cardiovascular death in heart failure patients or heart failure hospitalization are a reduced ejection fraction (for *EF* < 45%: 5% decrease in EF leads to 13% increase in hazard) and a higher NYHA class (with classes III and IV the hazard increases by 32 and 54% relative to patients in class II). In this study, the IHF_HR_ patient group was also characterized by a higher NYHA index and a reduced LVEF in relation to the IHF_LR_ patient group, reflecting the impairment of the physical performance and the left ventricular contractile function in high-risk patients. The biomarker NT-proBNP was dramatically increased in IHF_HR_ patients (the index with the highest significance being *p* = 2.7 × 10^−6^), confirming the role of NT-proBNP as one of the most useful indices for risk stratification of cardiac death in heart failure patients than other clinical indices (Bayes-Genis et al., 2007; Vazquez et al., 2009).

#### Non-clinical indices

Comparing the results of the univariate statistical analysis of non-clinical indices, we can conclude that indices from the linear domain and especially from nonlinear domain in particular from symbolic dynamics appear to be suitable for differentiation between low-risk and high-risk IHF patients, independent of the analyzed duration of NN interval time series and day phase. Only few non-clinical indices increased in value in the IHF_HR_ group when compared to the IHF_LR_ group; the majority of non-clinical indices decreased in the IHF_HR_ group, independent of the considered NN interval time series segments.

***Linear time and frequency domain HRV indices***. From the traditional HRV analysis (Task Force, 1996), meanNN of 24 h NN interval time series attained only negligible significant (0.0431) differences between IHF_HR_ and IHF_LR_. The analysis of the first 30 min segment (most comparable segment of all patients—the beginning of the ECG recording) revealed a significantly increased heart rate (*p* = 0.0165) in high-risk patients. An elevated resting heart rate was shown to be common in heart failure patients with systolic dysfunction and is an established marker of both morbidity and mortality risk (Cowie and Davidson, 2012). Increased heart rate in heart failure patients is associated with increased oxygen demand, reduced ventricular efficiency and relaxation, and atherogenesis. Furthermore, our study revealed a significantly lower sdNN in IHF_HR_ during nighttime. Thus, a reduced sdNN indicating decreased total variability seems to be suitable for detecting IHF_HR_ patients. Guzzetti et al. (Guzzetti et al., 2005b) demonstrated a decrease of nighttime sdNN but also of 24 h sdNN as predictors of non-sudden death in patients who died from progressive pump failure. Binici et al. (Binici et al., 2011) showed that a decreased nighttime sdNN in healthy subjects is associated with an increased stroke risk. Several studies (Ponikowski et al., 1997; Nolan et al., 1998; Galinier et al., 2000; Boveda et al., 2001; Bilchick et al., 2002) identified depressed sdNN of daytime and 24 h as predictive for all-cause mortality, heart failure death, and sudden death. With regard to the determined univariate significances (daytime: *p* = 0.1862 and 24 h: *p* = 0.1184) of sdNN differentiating between survival and cardiac death and the non-inclusion of sdNN into parameter sets using multivariate discriminant analysis, this finding could not be proven in our study. In all likelihood, unequal recording and analysis conditions including the length of the analyzed NN time series, the analyzed time of day, and physical activity during ECG recording could be responsible for inconsistent findings concerning sdNN. A reduction of the frequency domain index LF/HF seems to be useful for risk stratification of patients in the IHF_HR_ group. The highest significance (*p* = 0.0007) of LF/HF was obtained when analysing the 30 min most stationary day segment, and the lowest significance (*p* = 0.0186) was found when investigating the 30 min most stationary night segment. With regard to IHF_LR_ patients, a decrease of LF/HF in IHF_HR_ patients suggests a decreased cardiac sympathetic nerve activity and an increased vagal activity, and thus a considerable imbalance of the autonomous nervous system. An impaired sympathovagal balance can be confirmed by the normalized LF power (LFn) that was significantly lower (e.g., 30 min day segment, *p* = 0.0007) in IHF_HR_ (0.66 [0.44–0.75]) than in IHF_LR_ (0.76 [0.64–0.83]). The increase of vagal activity can be proven by the normalized HF power (HFn), reflecting mainly the respiratory sinus arrhythmia that was significantly increased (e.g., 30 min day segment, *p* = 0.0007) in the IHF_HR_ group [0.34 (0.25–0.56)] in relation to the IHF_LR_ group (0.24 [0.17–0.36]). LF/HF, LFn and HFn are calculated based on the frequency indices LF and HF. Their correlation is very strong (Pearson correlation coefficient in the range 74–85% depending on the analyzed ECG segment, *p* < 0.0001), and thus they are not independent. Therefore, LFn and HFn were excluded from the study and detailed information about both frequency indices are not provided in this paper. Several studies (Galinier et al., 2000; La Rovere et al., 2003; Guzzetti et al., 2005b) proved an advanced imbalance of the autonomous nervous system and demonstrated the usefulness of reduced LF power as a strong predictor of SCD and also of all-cause mortality in heart failure patients. Galinier et al. (Galinier et al., 2000) described a significant relationship between all-cause mortality and depressed LF power during daytime and nighttime periods. Furthermore, a significant relationship between low LF power during daytime and sudden death, and a significant association of low nighttime LF power with progressive heart failure death were shown. Later Guzzetti et al. (2005b) confirmed this finding, demonstrating that a reduction of LF power at night and an increased LVSD are linked to sudden mortality. La Rovere et al. (La Rovere et al., 2003) showed that a reduced LF power during controlled breathing calculated from 8 min ECG recordings in the morning predict sudden death with a relative risk of 2.8. In literature, the causes of a depressed LF power in heart failure are controversially discussed including abnormalities in the central autonomic regulation, depressed sinus node responsiveness, impaired baroreflex and beta-adrenergic receptor sensitivity, increased chemoreceptor sensitivity and a limitation in responsiveness to high cardiac sympathetic activation levels. As a further index from the frequency domain, VLF/P in IHF_HR_ patients was significantly reduced compared to IHF_LR_ patients during the most stationary phases of day and night periods. Guzzetti et al. (Guzzetti et al., 2005b) identified a reduced VLF power during nighttime HRV as being the strongest predictor of non-sudden death in patients who died from progressive pump failure. VLF oscillations are related to several slow regulatory mechanisms like physical activity, parasympathetic mechanisms, renin-angiotensin-aldosterone system, slow respiratory patterns and thermoregulation. Guzzetti et al. interpreted the reduction of VLF in heart failure patients that died from progressive pump failure to be the result of reduced physical activity in these more ill patients when compared to the surviving patients. He did not analyse the daytime HRV, but assumed that the relationship between VLF and the risk of progressive pump failure is an expression of a reduced physical activity, and thus VLF power during the day period as opposed to the night period should be the best predictor of death. When compared to our results, we can affirm this assumption: differences of VLF/P between low-risk and high-risk patients were more highly significant for the daytime period (*p* = 0.0026) than for the nighttime periods (*p* = 0.0444).

***Nonlinear HRV indices***. The largest amount of significant nonlinear HRV indices results from the calculation of indices of symbolic dynamics. From the classical SD method, wpsum13 and pW333 from 24 h and 30 min night HRV analysis were significantly reduced in IHF_HR_ compared to IHF_LR_, representing a lower portion of high variability patterns (wpsum13) in NN time series from high risk patients. This was particularly the case for patterns with three consecutive considerably shortened NN intervals when compared to meanNN (pW333) (Voss et al., 1996). During the night period, the probability of occurrence of word type “231” increased in the IHF_HR_ patient group. Additionally, an increase in probability of the intermittently low variability pattern plvar5 (successive NN differences <5 ms) in IHF_HR_ reflects a reduction of dynamics within the NN interval time series from high-risk IHF patients. The increase of plvar5 was especially pronounced in the most stationary night (*p* = 0.0052) and day (*p* = 0.0160) NN interval segments.

From STSD, m_ST_PEAK and m_ST_VAL significantly increased in the IHF_HR_ patient group when compared to IHF_LR_ during the following time segments: whole day (24 h, *p* < 0.05), the first 30 min of ECG recording (*p* < 0.01), and the 30 min night segment (*p* < 0.01). This means that the number of unstable patterns characterized by very fast changes of the NN intervals is increased in NN interval time series of high-risk patients, characterized by a decreased cardiac sympathetic nerve activity and an increased vagal activity (decreased LF/HF, decreased LFn, and increased HFn). Guzzetti et al. (Guzzetti et al., 2005a) also observed this effect under experimental and pharmacological conditions characterized by either sympathetic activation (tilt test, handgrip, nitroprusside, and high-dose atropine administration) or parasympathetic activation (phenylephrine and low-dose atropine administration). A parasympathetic prevalence induced an increase in ST_2V patterns (including m_ST_PEAK and m_ST_VAL), whereas an increase in sympathetic modulation and vagal withdrawal elicited a decrease in ST_2V pattern. In another study, Porta et al. (Porta et al., 2005) found that the number of unstable patterns (m_ST_PEAK and m_ST_VAL) significantly increased in heart failure patients when compared to healthy subjects, while the number of patterns with sustained changes (ST_ASC and ST_DESC) significantly decreased. In addition to significant increases of unstable patterns, we also found decreases of sustained patterns (only in trend) in high-risk heart failure patients when compared to low-risk patients. It is assumed that these findings are caused by increased electrical cardiac instabilities, resulting in more NN interval alternating patterns in heart failure patients (Porta et al., 2005). Furthermore, the portion of word patterns forming a plateau (s_ST_PLATEAU) significantly decreased in the IHF_HR_ group analysing the 24 h NN interval time series (*p* = 0.0099), indicating the loss of more stable patterns (s_ST_PLATEAU) in favor of more unstable patterns (m_ST_PEAK and m_ST_VAL).

A considerable depression of the SDSD index tau1_p001 (24 h, first 30 min, and 30 min night; *p* < 0.01) reflects a lowered mean short-term variability in conjunction with a lower complexity level in IHF_HR_ patients when compared to IHF_LR_ patients. In regard to low-risk IHF patients, a strongly diminished short-term scaling exponent α1 from DFA in IHF_HR_ patients indicates a lower portion of short-term correlations and a high degree of heart rate pattern randomness within BBI time series of high-risk IHF patients. A reduced α1 index in IHF_HR_ was observed for each statistical analysis regardless of the investigated NN interval duration (24 h or 30 min) and the analyzed NN interval segment (first 30 min, day- or night phase). Our results are in accordance with further research groups (Makikallio et al., 2001; Tapanainen et al., 2002; Stein et al., 2008; Salo et al., 2009), investigating several diseases and affirming that a decreased α1 exponent is suited to be an independent strong predictor of cardiac death, sudden death and total mortality. For example, Mäkikallio et al. (Makikallio et al., 2001) demonstrated that a reduced α1 index predicts cardiac death and SCD in heart failure with a relative risk of 2.5 and 4.1, respectively. A detailed physiological interpretation of the association of increased risk of death and decreased fractal correlation properties does not yet exist (Salo et al., 2009). Nevertheless, one possible explanation for a reduced α1 index in high-risk heart failure is the typical occurrence of high norepinephrine levels in such patients, indicating excessive sympathoexcitation. This probably leads to an increased randomness of heart rate behavior and consequentially to the reduction of α1 (Woo et al., 1994). The discrepancy between an increased sympathetic outflow (excessive sympathoexcitation) and a decreased LF/HF ratio is possibly caused by a feedback mechanism of circulating norepinephrine to sympathetic outflow, as described in Tulppo et al. (2005).

The significant decrease of SPPA_entropy in IHF_HR_ patients during the first 30 min of ECG recordings (*p* = 0.0024) when compared to IHF_LR_ patients reflects a loss of complexity and randomness in the HRV of high-risk patients. A decrease of SPPA_r_5 during the first 30 min of ECG recordings and an increase of SPPA_r_10 during the most stationary 30 min day segments in high-risk patients is a sign of an altered distribution of NN oscillations. SPPA_r_5 and SPPA_r_10 describe the probability distribution of occurring points in row 5 and row 10 of a 12 × 12-probability matrix, adjusted to the cloud of points of a Poincaré plot rotated 45 degrees (Voss et al., 2010a). Row 10 is located on the outer regions of the cloud (far from the plot's main focus) and indicates rapidly increased short-term variability of NN. Hence, an increase of SPPA_r_10 reflects more rapidly increasing short-term HRV patterns in IHF_HR_ patients during day segments when compared to IHF_LR_ patients. Row 5 is located in the inner regions of the cloud (near the main focus of the plot), therefore, a decrease of SPPA_r_5 indicates a reduction of slowly changing short-term HRV patterns in IHF_HR_ patients during the first 30 min of ECG recordings when compared to IHF_LR_ patients. From SSD (not calculable for 30 min time series), a reduction of s_pW111 indicates a lower variability of the word type “111” (three successive, considerably extended NN intervals) in IHF_HR_ patients than in IHF_LR_ patients. Considerably decreased Shannon entropies (Shannon_pW233, Shannon_pW332 and Shannon_pW333, *p* < 0.01) of distributions of the word types “233”, “332” and “333” were observed in IHF_HR_ when compared to IHF_LR_, reflecting more dominant peaks of that word types in the density distribution of IHF_HR_ patients. This indicates a more reduced single word variability within the windowed 24-h time series in high-risk patients. The word types “233”, “332” and “333” consisting of three consecutive shortened NN intervals when compared to the mean NN interval, are less complex and less random in IHF_HR_ as opposed to IHF_LR_. Decreased threshold-dependent indices m_pTH5, m_pTH6, m_pTH7 in IHF_HR_ are an expression of a lower number of highly frequent word types (probability thresholds of 5, 6, and 7%). The disappearance of some highly frequent word types leads to more dominant peaks in the density distribution of word types, indicating a lower complexity of HRV in IHF_HR_ when compared to IHF_LR_. A reduced variability and/or complexity of heart rate fluctuations could prove to be a significant predictor of mortality (Kleiger et al., 1987; Makikallio et al., 2001).

During this study, we also calculated indices by applying compression entropy, multiscale entropy, correlation function and mutual information analysis, as well as traditional Poincaré plot analysis. However, significant results when discriminating between low- and high risk IHF groups could not be proven.

### Multivariate analysis

When considering the multivariate classification results (Table 6) of the discriminant analysis, it could be shown that the best parameter set obtained from the first 30 min HRV analysis (mixed set 2 consisting of NT-proBNP, BMI, α1, SPPA_entropy, SPPA_r_5) revealed a slightly higher classification power (AUC = 84.3%) when compared to the following optimal mixed parameter sets: 24 h (AUC = 80.4%), 30 min day (AUC = 82.2%) and 30 min night (AUC = 81.7%). This indicates that 30 min HRV analysis may provide considerable risk stratification power due to the more standardized conditions, which exist during the initial 30 min recording time. With the exception of the optimal mixed set 1 attained for 24 h analysis, all mixed parameter sets (mixed sets 1 and 2) lead to an enhanced risk stratification as compared to the optimal clinical parameter set (increasing AUC from 76.9% to a maximum of 84.3%). Independent of the analyzed time segment (24 h and 30 min), the discrimination power of the best mixed parameter sets is similar (AUC range = 80.4–84.3%), and thus almost comparable. The optimal non-clinical parameter set obtained during analysis of the 24 h ECG showed nearly the same classification power (77.7%) as the optimal non-clinical parameter set obtained during the first 30 min ECG segment (AUC decrease = 0.1%), whereby the non-clinical sets determined from the 30min day and night segments revealed a lower discrimination power (AUC decrease approx. 6%). The classification results applying the optimal non-clinical parameter sets of 24 h and first 30 min analysis (AUCs 77.7 and 77.6%) and the clinical parameter set (AUC = 76.9%) are similar. The main advantage of a non-clinical parameter set as compared to a pure clinical parameter set is that linear and nonlinear HRV indices estimated from a conventional non-invasive ECG are not dependent on subjective influences and are easy to apply. Therefore, it could be possible to do risk stratification in IHF without complex, expensive (e.g., proBNP) and subjectively (NYHA) acquired clinical indices (Voss et al., 2010b). Otherwise, the combination of clinical and non-clinical parameters (mixed sets) leads to an improvement of risk stratification in IHF (AUC increase 3.5–7.4% when compared to the clinical parameter set). Hence, a careful selection of an appropriate mixed parameter set should be taken to achieve an optimal or acceptable balance between the cost and benefit of performing risk stratification. It should be noted that all clinical indices were ascertained by very experienced cardiologists, leading to an enhanced classification power of the clinical parameter set. An ascertainment of clinical indices by an inexperienced physician could considerably impair the classification power. Based on the results of discrimination power, the analysis of 24 h ECGs provides no benefit in the classification of high-risk IHF patients when compared to the analysis of 30 min ECGs. Therefore, 30 min ECG analysis seems to be sufficient for risk stratification in IHF.

In a subsequent study, the presented results need to be validated by analysing a larger amount of IHF patients, particularly of patients at high risk for cardiac death (possibly, after 5- or 10-year follow up periods). Furthermore, it would be interesting to investigate whether the same or similar parameter sets are suitable for enhanced risk stratification of sudden death or of total mortality in IHF or in other diseases.

In conclusion, the results of this study show that indices from frequency domain of HRV and nonlinear dynamics, particularly from symbolic dynamic, contribute (*p* < 0.05) to an enhanced risk stratification in IHF patients. Considerably changed indices in IHF_HR_ when compared to IHF_LR,_ indicate a higher vagal and a lower sympathetic modulation, a reduced HRV and a loss of complexity and randomness in HRV in IHF patients at high risk for a cardiac death. Indices from 24 h and 30 min time series, which were calculated by applying nonlinear HRV analysis methods, improved risk stratification in IHF patients as compared to a purely clinical parameter set. The classification power of optimal parameter sets estimated from the analysis of 24 h ECGs, the first 30 min of 24 h ECG recordings and of the 30 min most stationary day and night segment of the 24 h ECGs is almost comparable. Due to more standardized conditions during 30 min ECG recordings (particularly during the initial 30 min of the 24 h ECG recording) and due to the dramatically reduced time effort, short-term risk stratification in IHF seems to be an appropriate alternative to the Holter analysis. Hence, 30 min HRV analysis is most likely sufficient for risk stratification in IHF patients.

### Conflict of interest statement

The authors declare that the research was conducted in the absence of any commercial or financial relationships that could be construed as a potential conflict of interest.
